# Profile and Content of Phenolic Compounds in Leaves, Flowers, Roots, and Stalks of *Sanguisorba officinalis* L. Determined with the LC-DAD-ESI-QTOF-MS/MS Analysis and Their In Vitro Antioxidant, Antidiabetic, Antiproliferative Potency

**DOI:** 10.3390/ph13080191

**Published:** 2020-08-12

**Authors:** Sabina Lachowicz, Jan Oszmiański, Andrzej Rapak, Ireneusz Ochmian

**Affiliations:** 1 Department of Fermentation and Cereals Technology, Wrocław University of Environmental and Life Science, 51-630 Wrocław, Poland; 2 Department of Fruit, Vegetables and Nutraceutical Technology, Wrocław University of Environmental and Life Science, 51-630 Wroclaw, Poland; jan.oszmianski@upwr.edu.pl; 3 Laboratory of Tumor Molecular Immunobiology, Ludwik Hirszfeld Institute of Immunology and Experimental Therapy, Polish Academy of Sciences, 53-114 Wrocław, Poland; andrzej.rapak@hirszfeld.pl; 4 Department of Horticulture, West Pomeranian University of Technology in Szczecin, 71-434 Szczecin, Poland; ireneusz.ochmian@zut.edu.pl

**Keywords:** in vitro biological activity, bioactive compounds, morphological parts, medical plant

## Abstract

The aim of this study was to accurately determine the profile of polyphenols using the highly sensitive LC-DAD-ESI-QTOF-MS/MS technique and to determine in vitro antioxidant activity, the ability of inhibition of α-amylase, α-glucoamylase, and pancreatic lipase activity, and antiproliferative activity in leaves, flowers, roots, and stalks of medical plant *Sanguisorba officinalis* L. The results of the analysis of the morphological parts indicated the presence of 130 polyphenols, including 62 that were detected in *S. officinalis* L. for the first time. The prevailing group was tannins, with contents ranging from 66.4% of total polyphenols in the flowers to 43.3% in the stalks. The highest content of polyphenols was identified in the flowers and reached 14,444.97 mg/100 g d.b., while the lowest was noted in the stalks and reached 4606.33 mg/100 g d.b. In turn, the highest values of the antiradical and reducing capacities were determined in the leaves and reached 6.63 and 0.30 mmol TE/g d.b, respectively. In turn, a high ability to inhibit activities of α-amylase and α-glucoamylase was noted in the flowers, while a high ability to inhibit the activity of pancreatic lipase was demonstrated in the leaves of *S. officinalis* L. In addition, the leaves and the flowers showed the most effective antiproliferative properties in pancreatic ductal adenocarcinoma, colorectal adenocarcinoma, bladder cancer, and T-cell leukemia cells, whereas the weakest activity was noted in the stalks. Thus, the best dietetic material to be used when composing functional foods were the leaves and the flowers of *S. officinalis* L., while the roots and the stalks were equally valuable plant materials.

## 1. Introduction

The interest in alternative plants with a health-promoting potential has been growing in recent years not only in the pharmaceutical and cosmetic industries but also in the food industry where they are expected to contribute to the design of novel functional food. Therefore, it is believed that various morphological parts of *Sanguisorba officinalis* L. represent a good source of compounds exhibiting the aforementioned properties [1].

*S. officinalis* L. (great burnet or burnet bloodwort) is a species belonging to the Rosaceae family. It grows wild in Asia and Europe (except for the northern regions [1,2]. This melliferous, perennial plant usually occurs on arid and semi-arid grasslands and blooms from June till September. Its shoots can grow up to ca. 1.2–1.5 m. *S. officinalis* L. is resistant to frost as well as to diseases. It has been used for culinary purposes as an additive to salads and in animal feeding as an additive to feed mixtures due to its high nutritional value [3]. However, in folk medicine of both the Far East and Europe, *S. officinalis* L. was used as an herbal medicine in relieving inflammation, controlling external and internal bleeding, in the treatment of ulcers, burns, eczema, acne, as well as diarrhea [4,5]. In turn, the available experimental data prove a number of its biological properties, e.g., anti-inflammatory [3], anticancer [6], antiviral [7], antioxidant [1], prevention of the Alzheimer’s disease [3], and anti-wrinkle effects. [8]. In addition, the above studies have shown that all the biological properties exhibited by this perennial plant are due to a broad range of its bioactive compounds such as phenolic acids, tannins, flavonoids, triterpenes, and polysaccharides [1,3,4,5,6,7,8]. The richness of these compounds is sought in alternative plant sources that could be used in the treatment and prevention of many diseases and even as a dietary component [9].

Considering a number of biological properties of *S. officinalis* L., this plant has a high nutraceutical potential. However, there are a few reports on the profile and content of secondary metabolites in all of its morphological parts, which may differ and therefore exhibit various properties. Thus, research was undertaken into the accurate characterization of flowers, leaves, stalks, and roots in terms of the profile and content of polyphenols using the highly sensitive LC-DAD-ESI-QTOF-MS/MS technique. Analyses were also conducted to determine the in vitro antioxidant, antiproliferative, and antidiabetic activity for the individual morphological parts of *S. officinalis* L. This study aims to provide valuable information about differences in contents of bioactive compounds and their biological properties in the flowers, leaves, stalks, and roots of *S. officinalis* L., which will be used to compose not only functional foods but also nutraceuticals in the future.

## 2. Results and Discussion

### 2.1. Identification of Polyphenolic Compounds

The present study involved a thorough identification of the profile of bioactive compounds in extracts from leaves, flowers, stalks, and roots of *Sanguisorba officinalis* L. plant with the use of an ultrasensitive LC-DAD-ESI-QTOF-MS/MS method in the negative and positive ion mode. In total, 130 compounds were identified in extracts from the selected morphological parts of *S. officinalis* L., including 77 hydrolyzable tannins, 9 sanguiins, 3 sanguisorbic acids, 13 phenolic acids, 6 anthocyanins, 12 catechins and proanthocyanidins, and 9 flavonols, as well as 1 triterpenoid saponins (Table 1; Figures S1–S4). In turn, 62 compounds were identified in *S. officinalis* L. for the first time ever, including 42 hydrolyzable tannins, 5 sanguiins, 8 phenolic acids, 2 anthocyanins, 1 proanthocyanidins, and 3 flavonols as well as 1 triterpenoid saponins. Peaks were identified based on the determined exact molecular weights, peak retention times, primary ions from MS fragmentation, and comparison of data obtained with commercial standards and literature findings (Table 1). However, the profile of the compounds examined was strongly dependent on the morphological part of the plant, since 70, 76, 66, and 62 compounds were identified in the flowers, leaves, roots, and stalks, respectively.

The prevailing group of polyphenolic compounds were hydrolyzable tannins belonging to the family of tannins and being hydrolyzed conjugates that contain one or more hexahydroxydiphenoyl (HHDP) groups, thus leading to the esterification of sugars, glucose in particular. During fragmentation of the primary ions, losses observed were typical of these compounds and involved losses of galloyl, hexahydroxydiphenoyl, gallic acid, HHDP glucose, galloyl-glucose, and galloyl-HHDP-glucose residues with 152, 302, 170, 482, 332, 634 Da, respectively. Additionally, fragments were noted at *m*/*z* 169 and at *m*/*z* 301 formed through lactonization of the characteristic hexahydroxydiphenoyl group to ellagic acid. These compounds comprise typical galloyl and HHDP groups, respectively, which have earlier been described in the available literature [1,2,3,9,10,11]. Furthermore, if ellagitannin or galloyl derivates are composed of one or a few galloyl groups taking part in sugar synthesis, the fragmentary ion first discards a molecule of gallic acid and then a galloyl group or groups during fragmentation [10]. Among the 77 compounds, only 36 had previously been identified in *S. officinalis* L., and they all were methyl-6-*O*-galloyl-β-d-glucopyranoside (peak 17, 64; *m*/*z* 345), pedunculagin1 (18, 23, 29; *m*/*z* 785), galloyl-HHDP-glucose otherwise called corilagin isomer (25, 44, 55; *m*/*z* 633), di-galloyl-glucoside (37; *m*/*z* 483), methyl-4,6-digalloyl-*β*-d-glucopyranoside (50, 62, 71, 88; *m*/*z* 497), HHDP-galloyl-glucose (53; *m*/*z* 633), ellagic acid pentoside (60, 99; *m*/*z* 433), ellagic acid hexoside (67, 68, 102; *m*/*z* 463), di-galloyl hexoside (72, 118; *m*/*z* 483), galloyl-bis-HHDP-glucose otherwise called potentilin/casuarictin isomer (84, 85, 95, 97, 104, 106; *m*/*z* 935), lambertianin C (86; *m*/*z* 1401), ellagic acid (108; *m*/*z* 300.99), trigalloyl-HHDP-glucose (92, 114; *m/z* 937), trigalloyl-β-D-methyl glucoside (115; *m*/*z* 649), 3,3′,4′-*O*-trimethyl ellagic acid (127, 128; *m*/*z* 343), and 3,4′-*O*-dimethyl ellagic acid (129, 130; *m*/*z* 329) [2,3,12]. In turn, 16 compounds had earlier been detected and identified in flowers and fruits of *Punica granatum* but in this study were for the first time detected in the morphological parts of *S. officinalis* L. These compounds were referred to as: 2,3-HHDP-(α/β)-glucose (1; *m*/*z* 481), HHDP-hexoside(2,3-(*S*)-Hexahydroxydiphenoyl-d-glucose) (2, 4; *m*/*z* 481), HHDP-hexoside(1-galloyl-2,3-hexahydroxydiphenoyl-α-glucose) (3; *m*/*z* 481), galloyl-hexoside(β-glucogallin) (5; *m*/*z* 331), galloyl-hexoside (7–10, 13; *m*/*z* 331), di-HHDP-glucoside (34; *m*/*z* 783), di-galloyl-HHDP-glucose (14, 56, 66; *m*/*z* 785), galloyl-HHDP-hexoside (77; *m*/*z* 633), and pentagalloyl-glucoside (111; *m*/*z* 939) [10,13]. Another 6 compounds belonging to the group of hydrolyzable tannins were detected during identification of *Duchesnea indica* and they were: di-HHDP-glucose also known as pedunculalagin isomer (15, 20, 24, 26, 27, 30; *m*/*z* 783) [14]. However, 12 subsequent compounds were identified and determined based on their main ion and MS/MS fragmentation as β-1-*O*-galloyl-2,3-(*S*)-HHDP-d-glucose (28; *m*/*z* 633), methyl ellagic acid-pentoside (35; *m*/*z* 477), HHDP-NHTP-glucose (47, 51; *m*/*z* 933), castalagin/vescalagin isomer (58, 70, 79, 81, 98, 110; *m*/*z* 933), HHDP-NHTP-glucose-galloyl-di-HHDP-glucose (cocciferind2) (82; *m*/*z* 933), and tetragalloyl-glucose (100; *m*/*z* 787). They had earlier been detected in various plant materials like *Castanea sativa* Miller, *Quercus suber* L., *Betula pubescens*, raspberry fruits, and oak [15,16,17,18]. However, 8 compounds were identified for the first time ever. Compound No. 6 was tentatively identified as galloyl-pentoside based on the primary ion at *m*/*z* 301 and the loss of the pentose group (132 Da) giving a peak at *m*/*z* 169. Compound No. 49 was tentatively identified as HHDP-glucose based the primary ion at *m/z* 481 and MS/MS fragment at *m/z* 301. In the case of compound No. 54, the primary peak was at *m*/*z* at 345 due to the loss of a 176 Da residue that resulted in a peak formed at *m*/*z* 169, which was tentatively identified as galloyl-glucoronide. Compounds No. 73 and 74 were tentatively identified as eucaglobulin based on the primary ion at *m*/*z* 497 and MS/MS fragmentary ions revealing peaks at *m*/*z* 345, 327, 313, 183, and 169. In turn, compounds No. 93 and 94 were tentatively described as ellagic acid-hexoside-pentoside based the primary ion at *m*/*z* 595 and its fragmentation ions at *m*/*z* 433 and 301 due to the loss of a hexose residue (162 Da) and a pentose residue (132 Da). Finally, compound No. 113 was tentatively identified as methyl galloyl-glucoside based on the primary peak at *m*/*z* 345 and the loss of a glucosyl residue (162 Da), yielding a base peak at *m*/*z* 183.

Another described class of polyphenolic compounds belonging to hydrolyzed tannins were sanguiins. Among the 9 identified compounds, only 4 had earlier been detected in *S. officinalis* L. as sanguiin H-6 (11, 89; *m*/*z* 1870), sanguiin H-4 (41; *m*/*z* 633), and sanguiin H-10 isomer (48; *m*/*z* 783) by Karkanis et al. [3] and Zhu et al. [2], whereas the other 5 were never identified, as shown by literature data. Therefore, based on the primary peak at *m*/*z* 785 and MS/MS fragmentation peaks at *m*/*z* 633 and 301, and due to the loss of 152 and 332 Da groups, compounds No. 65, 69, and 96 were tentatively identified as sanguiin H-1. In turn, compounds No. 119 and 122 were tentatively identified as sanguiin H-7 and sanguiin H-7 isomers considering their primary ion at *m*/*z* 801 and fragmentation peaks at *m*/*z* 649 and 301 resulting from the loss of 152, 332, and 16 Da.

In contrast, sanguisorbic acids, belonging to the hydrolyzed tannins, also have been previously defined for these plants by Zhu et al. [2] as sanguisorbic acid dilactone (9, 12; *m*/*z* 469) and sanguisorbic acid glucoside (52; *m*/*z* 667). These compounds were determined only in the leaves, stalks, and roots of *S. officinalis* L. Moreover, 1 sanguisorbigenin, belonging to the triterpenoid saponins, was detected during identification *P. granatum* [12].

UV detection at the characteristic absorption maximum between 310 and 330 nm [19] showed the presence of 13 hydroxycinnamic acids in flowers, leaves, and stalks in the case of which the esterification of their quinic acid residue occurs at positions 3, 4, and 5, but not at position 1 [19]. Of these, 5 were identified early in *S. officinalis* as caffeoylquinic acid (16, *m*/*z* 353), 3-*O*-caffeoylquinic acid (19; *m*/*z* 353), 3-*O*-*p*-coumaroylquinic acid (32; *m*/*z* 337), 5-*O*-caffeoylquinic acid (42; *m*/*z* 353), and 3-*O*-feruloylquinic acid (78; *m*/*z* 367) [12]. However, 4 more were previously identified in other botanical sources like *Eryngium alpinum* L. and *Chrysanthemum* as rosmarinic acid (33; *m*/*z* 359), disuccinoyl-caffeoylquinic acids (116; *m*/*z* 553), and 3,5-dicaffeoylquinic (120, 121; *m*/*z* 515), however, for the first time in *S. officinalis* L., compounds No. 123–125 were tentatively identified as caffeoyl dihexoside based on the highest peak at *m*/*z* 505 and its fragmentation yielding peaks at *m*/*z* 341 and 179 due to the loss of 2 hexose residues (162 + 162 Da). What is more, these compounds were also described for the first time ever in morphological parts of *S. officinalis* L.

Anthocyanins are natural plant pigments occurring in the plant kingdom. They were identified in the positive ion mode because they bear a positive charge and easily donate protons to free radicals under ESI conditions. In turn, their detection was carried out at the typical absorption maximum between 440 and 540 nm [10,20]. Among the tentatively identified 6 anthocyanins, that were detected only in the flowers, only 4 were earlier determined in *S. officinalis* L. as cyanidin 3,5-diglucoside (21; *m*/*z* 611), cyanidin 3-*O*-glucoside (46; *m*/*z* 449), and cyanidin 3-malonylglucoside (76, 90; *m*/*z* 535) [12]. The other 2 compounds were described based on previous information about fragmentation of pomegranate and grape berry skin [13,21] as cyanidin 3-*O*-rutinoside (87; *m*/*z* 595) and cyanidin 3-(6-*O*-acetyl)-glucoside (91; *m*/*z* 491).

Flavan-3-ols occur as monomers, oligomers, and polymers formed by linking to (epi)catechin monomers via interflavonoid bonds (C–C) [22]. Their fragmentation proceeds through the loss of a (epi)catechin unit with a molecular weight of 289 Da. The identified proanthocyanins occurred as catechin dimers, trimers, and tetramers and were identified as A and B procyanidins [22]. These 11 compounds were characterized based on available standards and the latest research works addressing *S. officinalis* L as (+)-catechin and (−)-epicatechin (31, 40; *m*/*z* 289), B-type (epi)catechin dimmer (36, 38, 39, 63, 83; *m*/*z* 577), B-type (epi)catechin trimmer (43; *m*/*z* 865), and B-type (epi)catechin tetramer (57, 59, 80; *m*/*z* 1153) [2,3]. In turn, compound No. 74 was tentatively identified as a A-type (epi)catechin tetramer at *m*/*z* 1153 and the base ion at *m*/*z* 289. Although it was earlier detected in black soybean [23], it was described in *S. officinalis* L. for the first time ever.

Flavonols were identified as derivatives of taxifolin, kaempferol, and quercetin based on the base fragments at *m*/*z* 300, 285, and 301. UV detection of flavonols revealed characteristic absorption maximum between 315 and 359 nm, and some of the identified compounds had additional peaks between 207 and 280 nm [24]. Besides, derivatives of these compounds are usually detected at positions C-7 and/or C-3. Fragmentation of the primary ions resulted in losses of hexose (162 Da), pentose (146 Da), and deoxyhexose (308 Da) [24]. Of the 9 flavonols initially suggested for *S. officinalis* L., only 6 have previously been described for this species as quercetin-3-*O*-glucoside (45; 463), quercetin-3-*O*-(6″-galloylglucose) (101; *m*/*z* 615), taxifolin-7-*O*-*β*-d-glucopyranoside (103; *m*/*z* 465), quercetin-3-*O*-glucuronide (109; *m*/*z* 477), quercetin-3-*O*-acetyl glucoside (112; *m*/*z* 505), and kaempferol-3-*O*-glucuronide (117; *m*/*z* 461) [2,3,12]. In turn, 3 compounds have not been previously described according to the available literature. Compound No. 61 was tentatively identified as kaempferol-di-*O*-rhamnoside based on the primary peak at *m*/*z* 577 and fragmentation peaks at *m*/*z* 431 and 285 due to the loss of two rhamnoside residues (146 + 146 Da). Another compound (103) was tentatively described as quercetin-glucoside-dirhamnoside based on the primary peak at *m*/*z* 755 and fragmentation peaks at *m*/*z* 609, 463, and 301 due to the loss of two rhamnose residues and one glycosyl residue. Finally, compound No. 107 was tentatively presented as quercetin rhamnosyl-rutinoside based on the primary peak at *m*/*z* 755 and fragmentation peaks at *m*/*z* 609 and 301.

### 2.2. Quantification of Polyphenolic Compounds

The content of polyphenols in the analyzed morphological parts of *S. officinalis* L. is shown in Table 2. The highest content of bioactive compounds was determined in the flowers, it reached 14,444.97 mg/100 g d.b. and was 1.5, 1.7, and 3.2 times higher than in the leaves, roots, and stalks, respectively. In turn, the content of polyphenols in the leaves + stalks of *Sanguisorba minor* Scop. was comparable to the content of these compounds in *S. officinalis* L., while the roots of *S. minor* Scop. were 4 times more abundant in the studied compounds than the roots of *S. officinalis* L. [3]. In turn, the sum of polyphenols analysed in the roots of the same species from Korea was 2 times lower than in the roots of plants grown in Poland. However, the extract from *S. officinalis* L. cultivated in China contained 3150 mg GAE/100 g dry weight polyphenols, which was 4.9, 3.2, 2.8, and 1.5 times lower compared to the flowers, leaves, roots, and stalks of the same species growing in Poland. The content of polyphenols in the leaves of green and white tea was 67.21 and 40.94 mg/g d.b. and was 1.5 and 2.4 times lower than in the leaves of the studied species, respectively [25]. Total content of polyphenols analyzed in the flowers, leaves, roots, and stalks of *S. officinalis* L. was 8.2, 8.4, 7.8, and 8.4 times higher, respectively, compared to edible flowers of *Allium schoenoprasum* (Liliaceae), *Salvia pratensis* (Lamiaceae), *Sambucus nigra* (Caprifoliaceae), *Taraxacum officinale* [26]. However, according to Zeng et al. [27] the contents of bioactive compounds in the flowers of green and black tea of *Camellia sinensis* were 2.4 and 5.4 times lower, respectively, compared to the flowers of *S. officinalis* L. Moreover, the content of bioactive compounds in the flowers and the leaves of *Punica granatum* L. was 2.2 and 6.7 times lower, respectively, than in the same morphological parts of *S. officinalis* L. [28]. In addition, the content of compounds tested in the leaves and the stalks of *Fallopia japonica* was 1.7 and 2.3 times lower, respectively, while their content in the roots of *F. japonica* was similar to *S. officinalis* L. [9]. The differences in the contents of polyphenolic compounds among individual species can be affected by various factors, such as the place of cultivation, climate, environmental conditions, and also the method of extraction and analysis [29]. Thus, the tested material is characterized by a high content of compounds exhibiting a number of biological properties and can be used to compose not only nutraceuticals in the pharmaceutical industry but also to produce functional food.

The profile and content of phenols present in various morphological parts of *S. officinalis* L. were quite diverse and strongly dependent on the morphological part tested. The flowers were dominated by hydrolyzed tannins (66.4% in all phenols) > flavan-3-ols (13.1%) > phenolic acids (9.9%) > flavonols (5%) > anthocyanins (3.8%) > triterpenoids (1.8%). In turn, in the leaves were dominated by hydrolyzed tannins (49.3%) > phenolic acids (20.5%) > flavonols (19.8%) > flavan-3-ols (7.4%) > triterpenoids (3%). However, in the roots, hydrolyzed tannins were also the dominant class (62.1%) > flavan-3-ols (37.3%) > phenolic acids and flavonols (<0.5%), whereas the stalks were dominated by hydrolyzed tannins (43.3%) > flavan-3-ols (26.2%) > flavonols (17.1%) > phenolic acids (7.8%) > triterpenes (5.5%). The analysis of phenols profile revealed flavonols to be the major group in leaf + stalks, whereas hydrolyzed tannins to be the major group in the roots of *S. minor* [3], similarly to the roots of *S. officinalis* L. and to the results presented in the work of Kim et al. [1].

Tannins are compounds that occur naturally in plants and also play a defensive role in them. They exhibit anti-inflammatory properties against inflammation of the mucous membranes and skin, as well as antiastringent, antioxidative, free radical-scavenging, and antiproliferative properties. In addition, they are also an important component of food because they affect its storage stability, taste, and color [30]. The highest content of these compounds was recorded in the flowers (9594.27 mg/100 g d.b.) and the lowest one in the stalks (1996.51 mg/100 g d.b.). According to Karkanis et al. [3], their content in *S. minor* was comparable in the leaves and stalks while 4 times higher in the roots compared to the morphological parts of *S. officinalis* L., respectively. In turn, the major compound in all morphological parts tested was Lambertian C, with its content ranging from 62% in the roots to 17% in the stalks, and similar observations were made in *S. minor* [3].

Phenolic acids are another naturally occurring class of polyphenolic compounds that have a number of biological properties, including antioxidative ones, or are used in the prevention of cardiovascular diseases. They also affect the sour and bitter taste of food of plant origin, imparting them astringent flavones [31]. They dominated in the leaves of *S. officinalis* L. and their content amounted to 2044.37 mg/100 g d.b., while their poorest presence was in the roots (only 6.64 mg/100 g d.b.). Their content in the leaves was 5.3 times higher compared to their total content in leaves and stalks of *S. minor*, but similar while comparing to the stalks of *S. officinalis* L. and *S. minor* [3]. In turn, chlorogenic acid turned out to be the major compound in the flowers, neochlorogenic acid prevailed in the stalks and leaves, while ellagic acid was found in the leaves and stalks of *S. minor* [3].

Anthocyanins occurred only in flowers, giving them an intense red color. They belong to the group of polyphenols which show a number of health-promoting properties [9,32]. Their content was 549.57 mg/100 g d.b., and the dominant compounds were cyanidin 3-*O*-glucoside and cyanidin 3-*O*-malonylglucoside and they constituted of 62% and 28% of all anthocyanins, respectively.

Catechins and proanthocyanidins are compounds that also play an important role in the prevention of many diseases [9,32]. Their content ranged from 739.47 to 3239.19 mg/100 g d.b. in the leaves and roots of *S. officinalis* L, respectively, and was 5.6 and 20 times higher compared to the leaves and roots of *Fallopia japonica*, respectively [9]. The dominant compounds were: B-type (epi)catechin dimmer constituting 41% in the leaves to 18% in the stalks of all flavan-3-ols, and (−)-epicatechin constituting from 37% in the stalks to 19% in the leaves. Although in *F. japonica*, the major compound was procyanidin dimer B [9].

Flavonols are also a valuable class of natural secondary metabolites due to their anti-inflammatory and antioxidative properties [9]. The highest content of these compounds was noted in the leaves and reached 1969.85 mg/100 g d.b. It was 2.7, 2.5, and 41 times higher compared to the flowers, stalks, and roots, respectively. This difference results from the fact that these compounds are mainly located in the top layer of plants, protecting them from harmful UV radiation [32]. In turn, quercetin-*O*-glucuronide was the dominant compound in the flowers, leaves, and stalks, constituting 69%, 83%, and 85% of all flavonols, respectively, whereas taxifolin 7-*O*-β-d-glucopyranoside prevailed in the roots, constituting 91%. These observations have also been confirmed by Kim et al. [1].

### 2.3. Pro-Health Properties

The average antioxidative activity determined for *S. officinalis* L. was 4.45 mmol Troloxu (TE)/g dry basis (d.b.) in the ABTS test and 0.18 mmol TE/g d.b. in the FRAP assay (Table 3). The highest activity was determined in the leaves and was 6.63 and 0.30 mmol TE/g d.b. in the ABTS and FRAP tests, respectively. It was 1.2 and 1.6 times higher than in the stalks, 12.0 and 2.1 times higher than in the roots, and comparable to that found in the flowers for the ABTS radicals and for Fe^3+^ reduction to Fe^2+^, respectively (Table 3). Similar results of the antioxidative activity assays were obtained for the roots of *S. officinalis* gathered in China [5]. In turn, previous research shows that the antiradical activity of the leaves, stalks, and roots of *S. officinalis* L. was 6.2, 1.7, and 10.6 times higher compared to the same parts of *F. japonica* as well as 7.9, 1.8, and 9.3 times higher compared to the same parts of *F. sachalinensis*, respectively [9]. Antiradical activity for the roots was comparable to that obtained for the medical plant—*Ruta montana* [33]. Moreover, the average reducing activity of the tested parts of *S. officinalis* L. was comparable to the antioxidant potential determined for *Melissae* folium and about 6 times higher than for *Spiraea herba*, *Uvae ursi* folium, *Rubi fructose* folium, or *Fragariae herba* folium [34]. Thus, the results obtained indicate that the roots, flowers, and leaves of *S. officinalis* L. have a high ability to scavenge free radicals, which may be due to the high content of bioactive compounds determined for these morphological parts of the plant. What’s more, the results presented a strong Pearson’s correlation with the sum content of phenolic acids and anthocyanins and with the antioxidative activity as *r*^2^ = 0.734 and 0.539 for ABTS assay and *r*^2^ = 0.746 and 0.869 for FRAP, whereas the correlation between the reducing activity and sum of hydrolysable tannins and polyphenols was also strong *r*^2^ = 0.769 and 0.823.

The leaves, flowers, stalks, and roots of *S. officinalis* L. were also tested for their ability of inhibition of α-amylase (αA) and α-glucosidase (αG) activity, and their ability of inhibition of pancreatic lipase (LP) activity (Table 3). αA and αG are carbohydrate-degrading enzymes, but the mechanisms of their action differ; αA accelerates the hydrolysis of bonds inside a compound, whereas αG hydrolyzes α-1,4-glucosidic bonds, leading to the release of glucose absorbed by the body [35]. In turn, LP is an enzyme responsible for the degradation of triglycerides to simple lipids and fatty acids absorbable by the human body. However, it has been proved that excess fatty acids can lead to the formation of free radicals and insulin resistance [36]. Therefore, the inhibition of the above enzymes may be used in the treatment of diabetes type II or obesity [35]. The obtained results show that the highest ability to inhibit αA and αG activity was recorded for flowers of *S. officinalis* L. and reached EC_50_ 6.03 and 9.60 mg/mL, respectively. Therefore, the flowers were 1.6 and 1.3 times more active than the leaves, 4.0 and 3.3 times more active than the stalks, and 1.7 and 2.0 times more active than the roots, respectively. In turn, the highest ability to inhibit pancreatic lipase was found for the leaves of *S. officinalis* L. (EC_50_ = 18.75 mg/mL) which were 1.2, 3.0, and 3.9 times more active compared to the flowers, stalks, and roots of the tested plant, respectively. As far as the results showed that the ability to inhibit αA, αG, and LP strongly depended on the sum of flavan-3-ols and the correlations were *r*^2^ = 0.944, 0.836, and 0.593, respectively. However, in the case of phenolic acids and flavonols, the correlations were strongly negative: *r*^2^ = 0.813, 0.921, and 0.872 and *r*^2^ = 0.842, 0.825, and 0.857, respectively.

**Figure 1:**
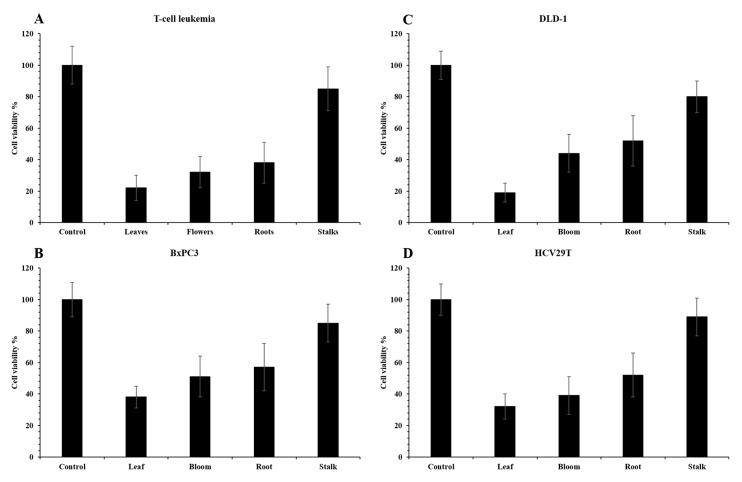
Cell viability of Jurkat (**A**), BxPC3 (**B**), DLD-1 (**C**), and HCV29T (**D**) cell lines after treatment with plant extracts for 48 h. Data are presented as means SD normalized to untreated control (1% ethanol).

The antiproliferative potency of the flowers, leaves, roots, and stems of *S. officinalis* L. were tested against four different cancer cell lines as BxPC3 (pancreatic ductal adenocarcinoma), DLD-1 (colorectal adenocarcinoma), HCV29T (bladder cancer), and Jurkat (T-cell leukemia). This is the first report on these cancer cell lines. The effect against the used cell lines was clearly noted (Figure 1). The extract from *S. officinalis* L. leaves significantly reduces the viability of all tested cell lines, especially DLD-1 colon cancer cells (to 19%) and Jurkat leukemia cells (to 22%). The flower extract reduced the viability of Jurkat cells to 32% and the remaining cells by 39–50%. Extract from the root showed similar results. In contrast, the extract from the stem acted the weakest on all cell lines, reducing cell viability to 85–97%. What’s more, the results presented a strong Pearson’s correlation between the sum of flavan-3-ols and with the viability of Jurkat leukemia cells and DLD-1 colon cancer cells—*r*^2^ = 0.731 and 0.545, while lower the viability of HCV29T cells strongly depended on anthocyanins and the correlation was *r*^2^ = 0.705. Liu et al. [37] noted that aqueous root extracts of *S. officinalis* L. showed synergic effect on inhibition of activity against HCT-116 and CPR cell lines (colon cancer) with 5-fluorouracil. Shin et al. [38] observed that the extract of *S. officinalis* L. inhibited cell growth against HSC4 and HN22 cell line (oral cancer) and induced death. According to Liu et al. [39], aqueous plant extracts of *S. officinalis* L. decreased the target Wnt and β-catenin genes by inhibiting the signal pathway of Wnt/β-catenin in cells of colorectal cancer. Moreover, Karkanis et al. [3] noted that the highest ability to inhibit of cervical carcinoma (HeLa), breast adenocarcinoma (MCF-7), and nonsmall cell lung cancer (NCl-H460) cell line was recorded for extract of roots of *S. minor*, whereas the extract of leaves + stalks of *S. minor* showed high ability to inhibit of hepatocellular carcinoma (HepG2) cell line. Thus, our own results and other authors presented that the highest cytotoxicity for the examined tumor cell lines covered depends on the analyzed morphological parts of *S. officinalis* L. and their bioactive substances. Moreover, the leaves, flowers, and roots showed high and differed antiproliferative potency to inhibit activity of various tumor cell lines.

## 3. Materials and Methods

### 3.1. Material, Reagents, and Instruments

Materials: *Sanguisorba officinalis* L. flowers, stalks, roots, and leaves (~5 kg) were obtained from a private garden in Szczytna (53°33′46″ N 20°59′07″ E), Lower Silesia, Poland. The plant was collected randomly in August 2019 from different parts of field (total area of cultivation is 1 ha). Then, material was washed and dried in a freeze dryer Alpha 1-4 LSC (Christ, Osterode, Germany).

Reagents: acetonitrile, formic acid, methanol, ABTS (2,2′-azinobis(3-ethylbenzothiazoline-6-sulfonic acid), 6-hydroxy-2,5,7,8-tetramethylchroman-2-carboxylic acid (Trolox), 2,4,6-tri(2-pyridyl)-s-triazine (TPTZ), methanol, acetic acid, α-amylase from porcine pancreas, α-glucoamylase from *Rhizopus* sp., lipase from porcine pancreas, Antibiotic-Antimycotic Solution, and RPMI 1640 culture medium were purchased from Sigma-Aldrich (Steinheim, Germany). (−)-Epicatechin, (+)-catechin, procyanidin B2, *p*-coumaric acid, ferulic acid, 5-caffeoylquinic acid, procyanidin A2, caffeic acid, quercetin 3-*O*-rutinoside, quercetin-3-*O*-galactoside, quercetin-3-*O*-glucoside, kaempferol 3-*O*-galactoside, ellagic acid, and cyanidin-3-*O*-glucoside were purchased from Extrasynthese (Lyon, France). DMEM culture medium with 10% FBS were purchased from Gibco (Thermo Fisher Scientific, Waltham, MA, USA), and MTS solution was purchased from Promega (Madison, WI, USA).

Instruments: UV-2401 PC spectrophotometer (Shimadzu, Kyoto, Japan) for antioxidant activity; Sonic 6D, Polsonic, Warsaw, Poland, for extraction; LC-DAD-ESI-QTOF-MS/MS (ultraperformance liquid chromatography equipped with a binary solvent manager and a Q-Tof Micro Mass Spectrometer (Waters, Manchester, UK) with an ESI source operating in negative and positive modes (Waters Corporation, Milford, MA, USA) for polyphenolic compounds; and Wallac 1420 VICTOR2 Plate Reader (PerkinElmer, Waltham, MA, USA) for antiproliferative activity.

### 3.2. Determination of Polyphenols

For the extraction and determination of phenolic compounds, a protocol described before by Lachowicz et al. [9] was followed. Briefly, samples (0.1 g) were mixed with 5 mL of 30% of UPLC-grade methanol. The extracts were sonicated for 20 min and centrifuged (at 19,000× *g*/10 min). Finally, the extracts were filtered by hydrophilic PTFE 0.20 μm membrane (Millex Samplicity Filter, Darmstadt, Germany) and used for testing.

The runs were monitored at the following wavelengths: phenolic acids at 320 nm, flavonols at 360 nm, anthocyanins at 520 nm, flavan-3-ols at 280 nm, and hydrolysable tannins at 240 nm. Separations of individual polyphenols were carried out using a UPLC BEH C18 column (1.7 μm, 2.1 mm × 100 mm) at 30 °C. The samples (10 μL) were injected, and the elution was completed in 15 min with a sequence of linear gradients and isocratic flow rates of 0.45 mL/min. The mobile phase consisted of solvent A (0.1% formic acid, v/v) and solvent B (100% acetonitrile). The program began with isocratic elution with 99% solvent A (0–1 min), and then, a linear gradient was used until 12 min, lowering solvent A to 0%; from 12.5 to 13.5 min, the gradient returned to the initial composition (99% A), and then, it was held constant to re-equilibrate the column. The analysis was carried out using full-scan, data-dependent MS scanning from *m*/*z* 100 to 1500. Leucine enkephalin was used as the reference compound at a concentration of 500 pg/μL, at a flow rate of 2 μL/min, and the [M − H]^−^ ion at 554.2615 Da was detected. The [M − H]^−^ ion was detected during 15 min analysis performed within ESI–MS accurate mass experiments, which were permanently introduced via the LockSpray channel using a Hamilton pump. The lock mass correction was ±1.000 for the mass window. The mass spectrometer was operated in negative- and positive-ion mode, set to the base peak intensity (BPI) chromatograms, and scaled to 12,400 counts per second (cps) (100%). The optimized MS conditions were as follows: capillary voltage of 2500 V, cone voltage of 30 V, source temperature of 100 °C, desolvation temperature of 300 °C, and desolvation gas (nitrogen) flow rate of 300 L/h. Collision-induced fragmentation experiments were performed using argon as the collision gas, with voltage ramping cycles from 0.3 to 2 V. Characterization of the single components was carried out via the retention time and the accurate molecular masses. Each compound was optimized to its estimated molecular mass [M − H]^−^/[M + H]^+^ in the negative and positive mode before and after fragmentation. The data obtained from UPLC-MS were subsequently entered into the MassLynx 4.0ChromaLynx Application Manager software. On the basis of these data, the software is able to scan different samples for the characterized substances. The PDA spectra were measured over the wavelength range of 200–800 nm in steps of 2 nm. The calibration curves were prepared for the standard: gallic acid (y = 1222.5x − 1972.7; *r*^2^ = 0.9999), procyanidin B2 (y = 6566.2x − 15,957; *r*^2^ = 0.9999), (+)-catechin (y = 1565.9x + 2243; *r*^2^ = 0.9999), *p*-coumaric acid (y = 68.109x + 49.224; *r*^2^ = 0.9996), ferulic acid (y = 50,215x + 36,206; *r*^2^ = 0.9997), 5-caffeoylquinic acid (y = 14,332x + 1315.1; *r*^2^ = 0.9999), procyanidin A2 (y = 9484.1x − 6770.5; *r*^2^ = 0.9997), caffeic acid (y = 17,431x + 40,114; *r*^2^ = 0.9999), quercetin 3-*O*-rutinoside (y = 13,362x − 1795; *r*^2^ = 0.9997), qercetin-3-*O*-galactoside (y = 20,926x − 18,309; *r*^2^ = 0.9991), qercetin-3-*O*-glucoside (y = 11,923x + 8188; *r*^2^ = 0.9999), kaempferol 3-*O*-galactoside (y = 12,057x − 1922.4; *r*^2^ = 0.9997), ellagic acid (y = 26754x + 172359; *r*^2^ = 0.9995), cyanidin-3-*O*-glucoside (y = 30,726x + 190,297; *r*^2^ = 0.9976), and (−)-epicatechin (y = 39,233x − 360,853; *r*^2^ = 0.9994) at concentrations ranging between 0.05 and 0.5 mg/mL. All data were obtained in triplicate. The results were expressed as mg/100 g of dry basis (d.b.).

### 3.3. Pro-Health Properties

#### 3.3.1. Antiradical Capacity

Samples (1 g) were mixed with methanol (80%; 10 mL) and then with hydrochloric acid (1%). This process was performed twice by incubating the above slurry for 20 min under sonication. Next, the slurry was centrifuged at 19,000× *g* for 10 min, and the supernatant was filtered through a hydrophilic PTFE 0.20 μm membrane (Merck, Darmstadt, Germany) and used for analysis.

The ABTS method was carried out with the method described by Re et al. [40]. For this, 0.03 mL of sample was mixed with 3 mL of ABTS + solution, and after 6 min of reaction, the absorbance was measured at 734 nm using the spectrophotometer. All data were obtained in triplicate. The activity was expressed in mmol Trolox/g d.b.

#### 3.3.2. Reducing Potential

The FRAP method was carried out with the method described by Benzie et al. [41]. The reagent was prepared by mixing 10 mmol 2,4,6-Tris(2-pyridyl)-s-triazine (TPTZ)/L reagent with 20 mmol/L ferric chloride in acetate buffer (pH 3.6). Precisely, 0.1 mL of sample was mixed with 0.9 mL of distilled water and 3 mL of ferric complex. After 10 min of reaction, the absorbance was measured at 593 nm using the spectrophotometer. All data were obtained in triplicate. The activity was expressed in mmol Trolox/g d.b.

#### 3.3.3. Determination of Enzyme Inhibition Potency

Anti-diabetic activity, α-amylase, α-glucosidase inhibitory, and lipase activity effect of the materials were described previously by Nakai et al. [42], Podsędek et al. [43], and Nickavar et al. [44]. The extraction of mixed material was done with 70% acetone (or water) at room temperature for 60 min with constant stirring. After centrifuging at 4000 rpm for 10 min, and filtration, the supernatants were concentrated at 40 °C (vacuum evaporator) to remove the acetone and the aqueous phase was diluted with water. For further analytical and biological activity assays, a gradient of concentrations was prepared via serial dilution of the fruit extracts in pure water. The amount of the inhibitor (expressed as mg of fruit per 1 mL of reaction mixture under assay conditions) required to inhibit 50% of the enzyme activity was defined as the IC_50_ value. The IC_50_ of the fruits tested was obtained from the line of the plot of the fruit concentration in 1 mL of reaction mixture versus the % inhibition. All samples were assayed in triplicate.

#### 3.3.4. Antiproliferative Potency

##### Cell Lines and Cell Culture

The human cancer cell lines BxPC3 (pancreatic ductal adenocarcinoma), DLD-1 (colorectal adenocarcinoma), and HCV29T (bladder cancer) were cultured in DMEM culture medium with 10% FBS and Antibiotic-Antimycotic Solution. Jurkat cell line (T-cell leukemia) was maintained in RPMI 1640 culture medium supplemented with 2 mM L-glutamine, 100 U/mL penicillin, 100 µg/mL streptomycin, and 10% fetal bovine serum (FBS). All cell lines were cultured at 37 °C in a humidified atmosphere of 5% CO_2_. The cells were seeded at densities of 5 × 10^3^ cells/0.1 mL (0.32 cm^2^) for cell viability assay. All cell lines were obtained from the collection of the Institute of Immunology and Experimental Therapy, Polish Academy of Sciences, Wroclaw, Poland.

##### Determination of Cell Viability

For determination of cell viability, cells were seeded in 96-well-plate (NUNC, Roskilde, Denmark). The plant extract was prepared by suspending 100 mg of dry plant material in 1 mL of 30% ethanol. The suspension was heated at 50 °C for 30 min and then centrifuged at 10,000× *g* for 15 min. The clear supernatant was diluted 30-fold in cell culture medium. As a control, 1% ethanol in the cell medium was used. The cells were incubated in 200 µL of the above culture medium for 48 h. Following the incubation, 20 µL of MTS solution was added to each well for 4 h; next, absorbance at 490 nm was recorded by a plate reader. Each treatment within a single experiment was performed in triplicate. Data were normalized to control medium containing 1% ethanol.

### 3.4. Statistical Analysis

Statistical analysis such as one-way ANOVA (*p* < 0.05) was analyzed using Statistica 12.5 (StatSoft, Kraków, Poland).

## 4. Conclusions

It needs to be noted that the flowers and leaves of *S. officinalis* L. are a good source of polyphenols, including hydrolyzable tannins, phenolic acids, flavonols, and anthocyanins, and exhibit a significant antiradical and reducing potential. In turn, the roots and stalks are a valuable source of flavan-3-ols. The most effective the inhibition of α-amylase, α-glucosidase, and pancreatic lipase and antiproliferative activities, reflected in the inhibition of viability of pancreatic ductal adenocarcinoma, colorectal adenocarcinoma, and bladder cancer as well as T-cell leukemia cell, were shown by the flowers and leaves of *S. officinalis* L. Thus, the data provided in this work indicate the possibility of using its individual morphological parts in the prevention of selected disease entities. In addition, this plant material can be used not only in the food industry as a functional additive to food, increasing its health value, but also in the cosmetic and pharmaceutical industries as a nutraceutical. The data obtained justify the need for further research on the morphological parts of *S. officinalis* L. with special emphasis put on leaves and flowers, to identify mechanisms potentially responsible for the antiproliferative activity.

## Figures and Tables

**Table 1:** Characterization of polyphenolic compounds in *Sanguisorba officinalis* L. by LC-DAD-ESI-QTOF-MS/MS.

No	Compounds	Rt [min]	Δ [nm]	MS/MS	F ^‡^	L	R	S
Hydrolyzable Tannins								
1	2,3-HHDP-(α/β)-glucose	1.31	272	481/463/301			x	
2	HHDP-hex(2,3-(*S*)-Hexahydroxydiphenoyl-d-glucose)	1.34	314	481/332/301/182	x	x	x	x
3	HHDP-hexoside(1-galloyl-2,3-hexahydroxydiphenoyl-α-glucose)	1.41	218	481/301/275/257/229		x		
4	HHDP-hex(2,3-(*S*)-Hexahydroxydiphenoyl-d-glucose)	1.50	314	481/330/306/301/203/182	x	x	x	x
5	Galloyl-hexoside(β-glucogallin)	1.86	278	331/169			x	
6	Galloyl-pentoside	1.99	274	301/169			x	
7	Galloyl-hexoside	2.08	272	331/169			x	
8	Galloyl-hexoside	2.09	268	331/169		x		
10	Galloyl-hexoside	2.52	278	331/169		x		x
13	Galloyl-hexoside	3.08	273	331/169		x		
14	Di-galloyl-HHDP-glucose (tellimagrandin I)	3.16	236/322	785/633/615/483/301	x	x		x
15	Di-HHDP-glucose (pedunculagin isomer)	3.34	230, 275 sh	783/481/301/257	x	x	x	x
17	Methyl-6-*O*-galloyl-β-D-glucopyranoside	3.54	274	345/169/124.99			x	x
18	Pedunculagin1	3.67	279	783/481/301	x			
20	Di-HHDP-glucose (pedunculagin isomer)	3.90	230, 275 sh	783/481/301/257	x			
23	Pedunculagin1	4.05	324	783/481/301	x			
24	Di-HHDP-glucose (pedunculagin isomer)	4.15	230, 275 sh	783/481/301/257	x			
25	Galloyl-HHDP-glucose (corilagin isomer)	4.18	235, 280 sh	633/300.99			x	
26	Di-HHDP-glucose (pedunculagin isomer)	4.24	326	783/481/301/257		x		
27	Di-HHDP-glucose (pedunculagin isomer)	4.24	230, 275 sh	783/481/301/257	x		x	
28	β-1-*O*-galloyl-2,3-(S)-HHDP-d-glucose	4.30	326	633/617/595/515/454/432/ 319/297/179	x	x		x
29	Pedunculagin1	4.30	279	783/481/301			x	
30	Di-HHDP-glucose (pedunculagin isomer)	4.40	313	783/613/447/423/274/211/ 196/169	x	x		x
34	Di-HHDP-glucoside	4.54	273	783/481/301			x	
35	Methylellagic acid-pentose	4.55	324	447/315/301	x	x		x
37	Di-galloyl-glucoside	4.59	273	483/313/169			x	
44	Galloyl-HHDP-glucose	4.98	219/276	633/463/301	x	x	x	x
47	HHDP-NHTP-glucose (castalagin/vescalagin)	5.08	219	933/915/889/871/631/613/587/569	x	x	x	x
49	HHDP-glucose	5.30	222	481/301	x	x	x	x
50	Methyl-4,6-digalloyl-β-d-glucopyranoside	5.39	212	497/345/169	x	x	x	x
51	HHDP-NHTP-glucose (castalagin/vescalagin)	5.44	282/343	933/915/889/871/631/613/587/569			x	
53	HHDP-galloyl-glucose	5.50	318	633/463/301/273/257/229/201/185	x			
54	Galloylglucoronide	5.52	276	345/169			x	
55	Galloyl-HHDP-glucose (corilagin isomer)	5.55	218	633/463/301		x		
56	Di-galloyl-HHDP-glucose (tellimagrandin I)	5.63	230, 280 sh	785/633/615/483/301	x	x		
58	Castalagin/vescalagin isomer	5.69	230, 285 sh	933/915/889/871/631/613/587/569	x	x		
60	Ellagic acid-pentoside	5.73	330	433/300.99	x	x		x
62	Methyl-4,6-digalloyl-β-d-glucopyranoside	5.90	216	497/345/169	x	x	x	x
64	Methyl-6-*O*-galloyl-β-d-glucopyranoside	5.97	374	345/169/124.99	x	x		x
66	Di-galloyl-HHDP-glucose (tellimagrandin I)	6.01	203/279	785/633/615/483/301			x	
67	Ellagic acid hexoside1	6.05	251/362	463/301	x	x	x	x
68	Ellagic acid hexoside	6.09	329	463/301				x
70	Castalagin/vescalagin isomer	6.15	230, 285 sh	933/915/889/871/631/613/587/569			x	
71	Methyl-4,6-digalloyl-β-D-glucopyranoside	6.19	213	497/345/169		x	x	x
72	Di-galloyl hexoside	6.22	203	483/301/169			x	
73	Eucaglobulin	6.23	276	497/345/327/313/183/169	x	x		x
75	Eucaglobulin	6.25	270	497/345/327/313/183/169	x	x		x
77	Galloyl-HHDP-hexoside	6.30	215	633/301			x	
79	Castalagin/vescalagin isomer	6.37	230, 285 sh	933/915/889/871/631/613/587/569	x	x	x	x
81	Castalagin/vescalagin isomer	6.41	222	933/915/889/871/631/613/587/569		x	x	x
82	HHDP-NHTP-glucose-galloyl-di-HHDP-glucose (cocciferind2)	6.46	224	933/915/633/631/301	x	x	x	x
84	Galloyl-bis-HHDP-glucose (potentilin/casuarictin isomer)	6.51	221	935/917/873//783/633/301	x	x	x	x
85	Galloyl-bis-HHDP-glucose (potentilin/casuarictin isomer)	6.55	225, 280 sh	935/917/873//783/633/301			x	
86	Lambertianin C	6.58	250	1401/1237/935/633303	x	x	x	x
88	Methyl-4,6-digalloyl-β-D-glucopyranoside	6.66	212	497/345/169			x	
92	Trigalloyl-HHDP-glucose	6.93	251 nm	937/767/635/465/301			x	
93	Ellagic acid-hexoside-pentoside	6.99	253/361	595/433/301	x	x	x	x
94	Ellagic acid-hexoside-pentoside	7.04	247/361	595/433/301		x		
95	Galloyl-bis-HHDP-glucose (potentilin/casuarictin isomer)	7.06	253/357	935/917/873//783/633/301			x	
97	Galloyl-bis-HHDP-glucose (potentilin/casuarictin isomer)	7.13	221	935/917/873//783/633/301	x			
98	Castalagin/vescalagin isomer	7.14	230, 285 sh	933/915/889/871/631/613/587/569				x
99	Ellagic acid pentoside	7.23	254/361	433/301	x	x	x	x
100	Tetragalloyl-glucose	7.27	227	787/635/617/573/465/403			x	
102	Ellagic acid hexoside	7.34	254/362	463/301	x	x	x	x
104	Galloyl-bis-HHDP-glucose (potentilin/casuarictin isomer)	7.41	218	935/917/873//783/633/301		x		
106	Galloyl-bis-HHDP-glucose (potentilin/casuarictin isomer)	7.43	219	935/917/873//783/633/301	x		x	
108	Ellagic acid ^a^	7.50	255/365	300.99	x	x	x	x
110	Castalagin/vescalagin isomer	7.81	250/373	933/915/889/871/631/613/587/569			x	
111	Pentagalloylglucoside	8.04	280	939/769/617/465/313/169			x	
113	Methyl galloyl-glucoside	8.24	297/325	345/183		x		
114	Trigalloyl-HHDP- glucose	8.26	259/360	937/7767/301			x	
115	Trigalloyl-β-*D*-methyl glucoside	8.35	263/356	649/497/479/345			x	
118	Di-galloyl hexoside	8.54	261/374	483/301			x	
127	3,3′,4′-*O*-trimethyl ellagic acid	9.66	352	343/328		x		
128	3,3′,4′-*O*-trimethyl ellagic acid	9.79	353	343/328		x		
129	3,4′-*O*-dimethyl ellagic acid	10.55	249/359	329/314/298/285			x	
130	3,4′-*O*-dimethyl ellagic acid	11.11	247/362	329/314/298/285			x	
Sanguiin								
11	Sanguiin H-6	2.74	234/320	1870/1567/1265/933/631/301	x	x		x
41	Sanguiin H-4	4.84	235/280 sh	633/300.99	x			
48	Sanguiin H-10 isomer	5.23	313	1567/1265/1103/933/301	x	x		x
65	Sanguiin H-1	5.99	230/280 sh	785/633/465/301	x			
69	Sanguiin H-1	6.13	254/371	785/633/465/301		x	x	x
89	Sanguiin H-6	6.75	236	1870/1567/1265/933/631/301	x	x	x	x
96	Sanguiin H-1	7.12	221	785/633/465/301		x	x	
119	Sanguiin H-7	8.59	261/361	801/649/301			x	
122	Sanguiin H-7 isomer	9.05	334	801/649/301	x	x		x
Sanguisorbic acids								
9	Sanguisorbic acid dilactone	2.13	272	469/314/301/286		x	x	
12	Sanguisorbic acid dilactone	2.89	275	469/314/301/286			x	
52	Sanguisorbic acid glucoside	5.47	325	667/285		x		x
Phenolic acids								
16	Caffeoylquinic acid ^a^	3.50	322	353/191/179/161	x	x		
19	3-*O*-caffeoylquinic acid ^a^	3.72	323	353/191/179/135	x	x		x
32	3-*O-p*-coumaroylquinic acid ^a^	4.50	311	337163	x	x		
33	Rosmarinic acid	4.54	325	359/191/179/173/163/152		x		x
42	5-*O*-caffeoylquinic acid ^a^	4.87	324	353/191/179	x	x		x
78	3-*O*-feruloylquinic acid ^a^	6.36	324	367/193/191	x	x		x
116	Disuccinoyl-caffeoylquinic acids	8.41	326	553/537/515/375/353/191/ 179/173	x	x		x
120	3,5-dicaffeoylquinic acid	8.83	326	515/353/191/179/173	x	x		x
121	3,5-dicaffeoylquinic acid	8.91	326	515/353/191/179/173	x	x		x
123	Caffeoyl dihexoside	9.27	325	503/341/179	x	x		x
124	Caffeoyl dihexoside	9.36	313	503/341/179	x	x		x
125	Caffeoyl dihexoside	9.50	326	503/341/179	x	x		x
126	Caffeoyl dihexoside	9.64	326	503/341/179			x	
Anthocyanins								
21	Cyanidin 3,5-*O*-diglucoside	3.91	520	611/449/287	x			
46	Cyanidin 3-*O*-glucoside ^a^	5.05	516	449/287	x			
76	Cyanidin 3-*O*-malonylglucoside	6.28	517	535/287	x			
87	Cyanidin 3-*O*-rutinoside	6.60	518	595/449/287	x			
90	Cyanidin 3-*O*-malonylglucoside	6.77	517	535/287	x			
91	Cyanidin 3-(6-*O*-acetyl)-glucoside	6.91	518	491/317/303/287	x			
Catechins and Proanthocyanidins								
31	(+)-Catechin ^a^	4.43	281	289	x	x	x	x
36	B-type (epi)catechin dimmer ^a^	4.58	276	577/289	x	x		x
38	B-type (epi)catechin dimmer ^a^	4.67	279	577/289		x	x	
39	B-type (epi)catechin dimmer ^a^	4.69	279	577/289	x	x		x
40	(−)-Epicatechin ^a^	4.83	279	289	x	x	x	x
43	B-type (epi)catechin trimmer	4.94	280	865/577/289				x
57	B-type (epi)catechin tetramer	5.63	278	1153/863/577/289	x	x	x	x
59	B-type (epi)catechin tetramer	5.70	278	1153/863/577/290	x	x	x	x
63	B-type (epi)catechin dimmer ^a^	5.90	274	577/289	x	x	x	x
74	A-type procyanidins tetramer	6.23	221/273	1153/865/575/			x	
80	B-type (epi)catechin tetramer	6.41	278	1153/863/577/289			x	
83	B-type (epi)catechin dimmer ^a^	6.46	276	577/289			x	
Flavonols								
45	Quercetin 3-*O*-glucoside ^a^	5.03	358	463/301		x		x
61	Kaempferol-di-*O*-rhamnoside	5.80	350	577/431/285	x	x		x
101	Quercetin 3-*O*-(6″-galloylglucose)	7.30	224	615/463/300.027		x		
103	Taxifolin 7-*O*-β-D-glucopyranoside	7.35	229	465/285			x	
105	Quercetin-glucoside-rhamnoside-rhamnoside	7.41	254/337	755/609/463/300.027	x	x		x
107	Quercetin rhamnosyl-rutinoside	7.47	368	755/609/301	x	x		x
109	Quercetin 3-*O*-glucuronide	7.68	255/353	477/300.027	x	x	x	x
112	Quercetin 3-*O*-acetyl glucoside	8.15	355	505/300.027	x	x		x
117	Kaempferol 3-*O*-glucuronide	8.49	347	461/285		x		x
Triterpenoid saponins								
22	Sanguisorbigenin	3.98	223/271	453/345/183/169	x	x		x

^‡^ F, flowers; L, leaves; R, roots; S, stalks; ^a^ identification confirmed by commercial standards.

**Table 2:** Content of polyphenolic compounds in *Sanguisorba officinalis* [mg/100 g d.w.].

	Compounds	Flower	Leaves	Roots	Stalk
	Hydrolyzable tannins				
1	2,3-HHDP-(α/β)-glucose	nd ^‡^	nd	12.33 ± 0.25a ^†^	nd
2	HHDP-hex(2,3-(*S*)-Hexahydroxydiphenoyl-d-glucose)	141.89 ± 2.84a	102.71 ± 2.05b	13.28 ± 0.27c	11.49 ± 0.23c
3	HHDP-hexoside(1-galloyl-2,3-hexahydroxydiphenoyl-α-glucose)	nd	14.36 ± 0.29a	nd	nd
4	HHDP-hex(2,3-(*S*)-Hexahydroxydiphenoyl-d-glucose)	161.00 ± 3.22a	63.35 ± 1.27b	40.73 ± 0.81c	12.49 ± 0.25d
5	Galloyl-hexoside(β-glucogallin)	nd	nd	92.13±1.84a	nd
6	Galloyl-pentoside	nd	nd	38.51±0.77a	nd
7	Galloyl-hexoside	nd	nd	20.66±0.41a	nd
8	Galloyl-hexoside	nd	13.89 ± 0.28a	nd	nd
10	Galloyl-hexoside	nd	5.18 ± 0.10b	nd	9.52 ± 0.19a
13	Galloyl-hexoside	nd	4.41 ± 0.09a	nd	nd
14	Di-galloyl-HHDP-glucose (tellimagrandin I)	5.57 ± 0.11a	6.34 ± 0.13a	nd	1.35 ± 0.03b
15	Di-HHDP-glucose (pedunculagin isomer)	100.66 ± 2.01b	24.25 ± 0.49c	136.03 ± 2.72a	15.78 ± 0.32d
17	Methyl-6-*O*-galloyl-β-D-glucopyranoside	nd	nd	234.27 ± 4.69a	7.20 ± 0.14b
18	Pedunculagin1	2.55 ± 0.05a	nd	nd	nd
20	Di-HHDP-glucose (pedunculagin isomer)	2.23 ± 0.04a	nd	nd	nd
23	Pedunculagin1	9.08 ± 0.18a	nd	nd	nd
24	Di-HHDP-glucose (pedunculagin isomer)	20.00 ± 0.40a	nd	nd	nd
25	Galloyl-HHDP-glucose (corilagin isomer)	nd	nd	29.73 ± 0.59a	nd
26	Di-HHDP-glucose (pedunculagin isomer)	nd	17.21 ± 0.34a	nd	nd
27	Di-HHDP-glucose (pedunculagin isomer)	97.32 ± 1.95a	nd	42.58 ± 0.85b	nd
28	β-1-*O*-galloyl-2,3-(S)-HHDP-d-glucose	513.20 ± 10.26a	433.89±8.68b	nd	83.52 ± 1.67c
29	Pedunculagin1	nd	nd	24.37 ± 0.49a	nd
30	Di-HHDP-glucose (pedunculagin isomer)	9.66 ± 0.19b	11.96 ± 0.24a	nd	2.01 ± 0.04c
34	Di-HHDP-glucoside	nd	nd	19.51 ± 0.39a	0
35	Methylellagic acid-pentose	26.83 ± 0.54a	5.45 ± 0.11c	nd	8.17 ± 0.16b
37	Di-galloyl-glucoside	nd	nd	53.85 ± 1.08a	nd
44	Galloyl-HHDP-glucose	165.31 ± 3.31a	8.65 ± 0.17c	145.15 ± 2.90b	5.25 ± 0.11d
47	HHDP-NHTP-glucose (castalagin/vescalagin)	87.29 ± 1.75b	100.59 ± 2.01a	41.30 ± 0.83c	23.36 ± 0.47d
49	HHDP-glucose	97.26 ± 1.95a	45.3 ± 0.91b	11.32 ± 0.23c	11.44 ± 0.23c
50	Methyl-4,6-digalloyl-β-d-glucopyranoside	7.94 ± 0.16b	1.06 ± 0.02c	17.12 ± 0.34a	0.58 ± 0.01d
51	HHDP-NHTP-glucose (castalagin/vescalagin)	nd	nd	24.08 ± 0.48a	nd
53	HHDP-galloyl-glucose	43.97 ± 0.88a	nd	nd	nd
54	Galloylglucoronide	nd	nd	93.44 ± 1.87a	nd
55	Galloyl-HHDP-glucose (corilagin isomer)	nd	22.90 ± 0.46a	nd	nd
56	Di-galloyl-HHDP-glucose (tellimagrandin I)	85.77 ± 1.72a	35.62 ± 0.71b	nd	nd
58	Castalagin/vescalagin isomer	37.38 ± 0.75a	70.71 ± 1.41b	nd	nd
60	Ellagic acid-pentoside	9.31 ± 0.19b	13.70 ± 0.27a	nd	3.96 ± 0.08c
62	Methyl-4,6-digalloyl-β-d-glucopyranoside	256.75 ± 5.14a	104.29 ± 2.09b	254.04 ± 5.08a	71.93 ± 1.44c
64	Methyl-6-*O*-galloyl-β-d-glucopyranoside	6.75 ± 0.14b	10.71 ± 0.21a	nd	3.47 ± 0.07c
66	Di-galloyl-HHDP-glucose (tellimagrandin I)	nd	nd	13.52 ± 0.27a	nd
67	Ellagic acid hexoside	5.76 ± 0.12b	7.16 ± 0.14a	4.05 ± 0.08b	2.61 ± 0.05c
68	Ellagic acid hexoside	nd	nd	nd	4.53 ± 0.09a
70	Castalagin/vescalagin isomer	nd	nd	68.46 ± 1.37a	nd
71	Methyl-4,6-digalloyl-β-D-glucopyranoside	nd	1.80 ± 0.04a	1.70 ± 0.03a	0.58 ± 0.01b
72	Di-galloyl hexoside	nd	nd	43.6±0.87a	nd
73	Eucaglobulin	51.84 ± 1.04b	102.83 ± 2.06a	nd	16.79 ± 0.34c
75	Eucaglobulin	71.19 ± 1.42a	71.72 ± 1.43a	nd	22.59 ± 0.45b
77	Galloyl-HHDP-hexoside	nd	nd	106.23 ± 2.12a	nd
79	Castalagin/vescalagin isomer	26.13 ± 0.52c	62.30 ± 1.25a	52.75 ± 1.06b	14.52 ± 0.29d
81	Castalagin/vescalagin isomer	nd	92.82 ± 1.86a	67.43 ± 1.35b	13.19 ± 0.26c
82	HHDP-NHTP-glucose-galloyl-di-HHDP-glucose (cocciferind2)	87.01 ± 1.74b	41.02 ± 0.82c	155.76 ± 3.12a	13.57 ± 0.27d
84	Galloyl-bis-HHDP-glucose (potentilin/casuarictin isomer)	38.45 ± 0.77b	132.33 ± 2.65a	32.87 ± 0.66c	30.56 ± 0.61c
85	Galloyl-bis-HHDP-glucose (potentilin/casuarictin isomer)	nd	nd	52.26 ± 1.05a	nd
86	Lambertianin C	3029.28 ± 60.59a	2232.84 ± 44.66b	898.98 ± 17.98d	1236.77 ± 24.74c
88	Methyl-4,6-digalloyl-β-D-glucopyranoside	nd	nd	4.82 ± 0.1a	nd
92	Trigalloyl-HHDP-glucose	nd	nd	86.34 ± 1.73a	nd
93	Ellagic acid-hexoside-pentoside	33.54 ± 0.67a	32.53 ± 0.65a	32.80 ± 0.66a	7.09 ± 0.14b
94	Ellagic acid-hexoside-pentoside	nd	51.34 ± 1.03a	nd	nd
95	Galloyl-bis-HHDP-glucose (potentilin/casuarictin isomer)	nd	nd	12.48 ± 0.25a	nd
97	Galloyl-bis-HHDP-glucose (potentilin/casuarictin isomer)	30.53 ± 0.61a	nd	nd	nd
98	Castalagin/vescalagin isomer	nd	nd	nd	43.38 ± 0.87a
99	Ellagic acid pentoside	14.50 ± 0.29b	15.22 ± 0.3b	18.07 ± 0.36a	3.47 ± 0.07c
100	Tetragalloyl-glucose	nd	nd	328.94 ± 6.58a	nd
102	Ellagic acid hexoside1	1.14 ± 0.02a	0.33 ± 0.01c	0.61 ± 0.01b	0.36 ± 0.01c
104	Galloyl-bis-HHDP-glucose (potentilin/casuarictin isomer)	nd	56.41 ± 1.13a	nd	nd
106	Galloyl-bis-HHDP-glucose (potentilin/casuarictin isomer)	202.46 ± 4.05a	nd	147.72 ± 2.95b	nd
108	Ellagic acid	17.69 ± 0.35c	26.90 ± 0.54a	13.49 ± 0.27b	5.20 ± 0.10d
110	Castalagin/vescalagin isomer	nd	nd	1.91 ± 0.04a	nd
111	Pentagalloylglucoside	nd	nd	36.57 ± 0.73a	nd
113	Methyl galloyl-glucoside	nd	13.75 ± 0.28a	nd	nd
114	Trigalloyl-HHDP- glucose	nd	nd	0.71 ± 0.01a	nd
115	Trigalloyl-β-*D*-methyl glucoside	nd	nd	35.65 ± 0.71a	nd
118	Di-galloyl hexoside	nd	nd	3.61 ± 0.07a	nd
127	3,3′,4′-*O*-trimethyl ellagic acid	nd	31.41 ± 0.63a	nd	nd
128	3,3′,4′-*O*-trimethyl ellagic acid	nd	1.47 ± 0.03a	nd	nd
129	3,4′-*O*-dimethyl ellagic acid	nd	nd	49.05 ± 0.98a	nd
130	3,4′-*O*-dimethyl ellagic acid	nd	nd	251.11 ± 5.02a	nd
	SUM	5497.24 ± 109.94a	4090.71 ± 81.81b	3865.92 ± 77.32c	1686.73 ± 33.73d
	Sanguiin				
11	Sanguiin H-6	2.57 ± 0.05b	10.13 ± 0.20a	nd	1.22 ± 0.02c
41	Sanguiin H-4	352.14 ± 7.04a	nd	nd	nd
48	Sanguiin H-10 isomer	130.92 ± 2.62a	5.33 ± 0.11b	nd	4.14 ± 0.08b
65	Sanguiin H-1	43.36 ± 0.87	nd	nd	nd
69	Sanguiin H-1	nd	1.01 ± 0.02b	2.95 ± 0.06a	0.15 ± 0.01c
89	Sanguiin H-6	3566.15 ± 71.32a	621.04 ± 12.42d	763.91 ± 15.28c	289.86 ± 5.80b
96	Sanguiin H-1	nd	61.95 ± 1.24b	730.22 ± 14.60a	nd
119	Sanguiin H-7	nd	nd	4.42 ± 0.09a	nd
122	Sanguiin H-7 isomer	1.89 ± 0.04a	2.24 ± 0.04a	nd	0.98 ± 0.02b
	SUM	4097.03 ± 81.94a	701.7 ± 14.03c	1501.5 ± 30.03b	296.35 ± 5.93d
	Sanguisorbic acids				
9	Sanguisorbic acid dilactone	nd	6.61 ± 0.13d	10.95 ± 0.22a	nd
12	Sanguisorbic acid dilactone	nd	nd	15.44 ± 0.31a	nd
52	Sanguisorbic acid glucoside	nd	109.18 ± 2.18a	nd	13.43 ± 0.27b
	SUM	nd	115.79 ± 2.32a	26.39 ± 0.53b	13.43 ± 0.27c
	Phenolic acids				
16	Caffeoylquinic acid	23.07 ± 0.46b	47.52 ± 0.95a	nd	nd
19	Caffeoylquinic acid	539.00 ± 10.78b	1363.67 ± 27.27a	nd	182.92 ± 3.66c
32	3-*p*-Coumaroylquinic acid	87.17 ± 1.74a	42.55 ± 0.85b	nd	nd
33	Rosmarinic acid	nd	8.39 ± 0.17a	nd	2.98 ± 0.06b
42	5-Caffeoylquinic acid	673.42 ± 13.47a	436.44 ± 8.73b	nd	129.09 ± 2.58c
78	3-Feruloylquinic acid	11.46 ± 0.23a	4.95 ± 0.10b	nd	3.17 ± 0.06c
116	Disuccinoyl-caffeoylquinic acids	69.02 ± 1.38b	89.00 ± 1.78a	nd	31.51 ± 0.63c
120	Di-caffeoylquinic	4.81 ± 0.10b	17.66 ± 0.35a	nd	2.79 ± 0.06c
121	Dicaffeoylquinic	4.12 ± 0.08c	12.78 ± 0.26a	nd	1.33 ± 0.03c
123	Caffeoyl dihexoside	2.72 ± 0.05b	6.68 ± 0.13a	nd	3.10 ± 0.06b
124	Caffeoyl dihexoside	13.38 ± 0.27a	8.47 ± 0.17b	nd	2.04 ± 0.04c
125	Caffeoyl dihexoside	3.51 ± 0.07b	6.26 ± 0.13a	nd	2.23 ± 0.04c
126	Caffeoyl dihexoside	nd	nd	6.64 ± 0.13a	nd
	SUM	1431.68 ± 28.63b	2044.37 ± 40.89a	6.64 ± 0.13d	361.16 ± 7.22c
	Anthocyanins				
21	Cyanidin 3,5-*O*-diglucoside	19.56 ± 0.39a	nd	nd	nd
46	Cyanidin 3-*O*-glucoside	339.87 ± 6.80a	nd	nd	nd
76	Cyanidin 3-*O*-malonylglucoside	154.35 ± 3.09a	nd	nd	nd
87	Cyanidin 3-*O*-rutinoside	4.83 ± 0.10a	nd	nd	nd
90	Cyanidin 3-*O*-malonylglucoside	14.40 ± 0.29a	nd	nd	nd
91	Cyanidin 3-(6-*O*-acetyl)glucoside	16.56 ± 0.33a	nd	nd	nd
	SUM	549.57 ± 10.99a	nd	nd	nd
	Catechins and Proanthocyanins				
31	(+)-Catechin	46.77 ± 0.94d	160.08 ± 3.20b	374.41 ± 7.49a	133.37 ± 2.67c
36	B-type (epi)catechin dimmer	111.05 ± 2.22a	33.03 ± 0.66b	nd	28.85 ± 0.58c
38	B-type (epi)catechin dimmer	nd	19.88 ± 0.40b	383.49 ± 7.67a	nd
39	B-type (epi)catechin dimmer	136.33 ± 2.73a	15.04 ± 0.30c	nd	125.77 ± 2.52b
40	(−)-Epicatechin	656.57 ± 13.13b	138.19 ± 2.76d	700.12 ± 14.00a	457.66 ± 9.15c
43	B-type (epi)catechin trimmer	nd	nd	nd	86.20 ± 1.72a
57	B-type (epi)catechin tetramer	120.62 ± 2.41c	45.32 ± 0.91d	448.56 ± 8.97a	142.85 ± 2.86b
59	B-type (epi)catechin tetramer	57.12 ± 1.14a	22.38 ± 0.45b	21.69 ± 0.43b	18.43 ± 0.37c
63	B-type (epi)catechin dimmer	760.26 ± 15.21b	305.55 ± 6.11c	796.86 ± 15.94a	214.39 ± 4.29d
74	A-type procyanidin tetramer	nd	nd	51.53 ± 1.03a	nd
80	B-type (epi)catechin tetramer	nd	nd	105.67 ± 2.11a	nd
83	B-type (epi)catechin dimmer	nd	nd	356.86 ± 7.14a	nd
	SUM	1888.72 ± 37.77b	739.47 ± 14.79d	3239.19 ± 64.78a	1207.52 ± 24.15c
	Flavonols				
45	Quercetin 3-*O*-glucoside	nd	15.00 ± 0.30a	nd	4.15 ± 0.08b
61	Kaempferol-di-*O*-rhamnoside	5.23±0.10a	0.59 ± 0.01b	nd	0.31 ± 0.01b
101	Quercetin 3-*O*-(6″-galloylglucose)	nd	77.72 ± 1.55a	nd	nd
103	Taxifolin 7-*O*-β-D-glucopyranoside	nd	nd	43.41 ± 0.87a	nd
105	Quercetin-glucoside-rhamnoside-rhamnoside	26.29 ± 0.53a	9.93 ± 0.20c	nd	13.33 ± 0.27b
107	Quercetin rhamnosyl-rutinoside	5.93 ± 0.12a	3.11 ± 0.06b	nd	2.54 ± 0.05b
109	Quercetin 3-*O*-glucuronide	494.97 ± 9.90c	1645.76 ± 32.92a	4.13 ± 0.08d	675.15 ± 13.50b
112	Quercetin 3-*O*-acetyl glucoside	47.89 ± 0.96b	54.56 ± 1.09a	nd	26.73 ± 0.53c
117	Kaempferol 3-*O*-glucuronide	137.89 ± 2.76b	163.18 ± 3.26a	nd	65.65 ± 1.31c
	SUM	718.2 ± 14.36c	1969.85 ± 39.40a	47.54 ± 0.95d	787.86 ± 15.76b
	Sanguisorbigenin	262.53 ± 5.25b	300.60 ± 6.01a	nd	253.28 ± 5.07c
	Total mg/100 g d.w.	14444.97 ± 288.90a	9962.55 ± 199.25b	8687.16 ± 173.74c	4606.33 ± 92.13d

^†^ Values are expressed as the mean (*n* = 3) ± standard deviation and different letters (between morphological parts) within the same row indicates statistically significant differences (*p* < 0.05); ^‡^ nd, not identified.

**Table 3:** The antioxidant activity and the biological activity in vitro.

Components	α-Amylase [EC_50_ MG/ML]	α-Glucosidase [EC_50_ MG/ML]	Pancreatic Lipase [EC_50_ MG/ML]	ABTS [mmol/g d.b.]	FRAP [mmol/g d.b.]
Leaves	9.48 ± 0.24b ^‡^	11.86 ± 0.24b	18.75 ± 0.38a	6.63 ± 0.1a3	0.30 ± 0.01a
Flowers	6.03 ± 0.19a	9.60 ± 0.19a	21.40 ± 0.43b	5.56 ± 0.11b	0.20 ± 0.01b
Stalks	23.91 ± 0.63c	31.74 ± 0.63d	56.47 ± 1.13c	0.52 ± 0.01d	0.09 ± 0.01d
Roots	10.44 ± 0.39b	19.54 ± 0.39c	72.68 ± 1.45d	5.08 ± 0.10c	0.13 ± 0.01c

^‡^ Values are expressed as the mean (*n* = 3) ± standard deviation and different letters (between morphological parts) within the same row indicates statistically significant differences (*p* < 0.05).

## Supplementary Materials

The following are available online at https://www.mdpi.com/1424-8247/13/8/191/s1. Figure S1: LC-DAD-ESI-QTOF-MS/MS chromatogram fragile of the *Sanguisorba officinalis* L. flowers extract at 320 and 360 nm; Figure S2: LC-DAD-ESI-QTOF-MS/MS chromatogram fragile of the *Sanguisorba officinalis* L. leaves extract at 320 and 360 nm; Figure S3: LC-DAD-ESI-QTOF-MS/MS chromatogram fragile of the *Sanguisorba officinalis* L. roots extract at 320 and 360 nm; Figure S4: LC-DAD-ESI-QTOF-MS/MS chromatogram fragile of the *Sanguisorba officinalis* L. stalks extract at 320 and 360 nm.
