# Supplementary material for: Profile and Content of Phenolic Compounds in Leaves, Flowers, Roots, and Stalks of Sanguisorba officinalis L. Determined with the LC-DAD-ESI-QTOF-MS/MS Analysis and Their In Vitro Antioxidant, Antidiabetic, Antiproliferative Potency
Source: Pharmaceuticals (Basel). 2020 Aug 12;13(8):191. doi: 10.3390/ph13080191 (PMC7464974; doi:10.3390/ph13080191)
Supplement: Supplementary file 1 [file pharmaceuticals-13-00191-s001.pdf]

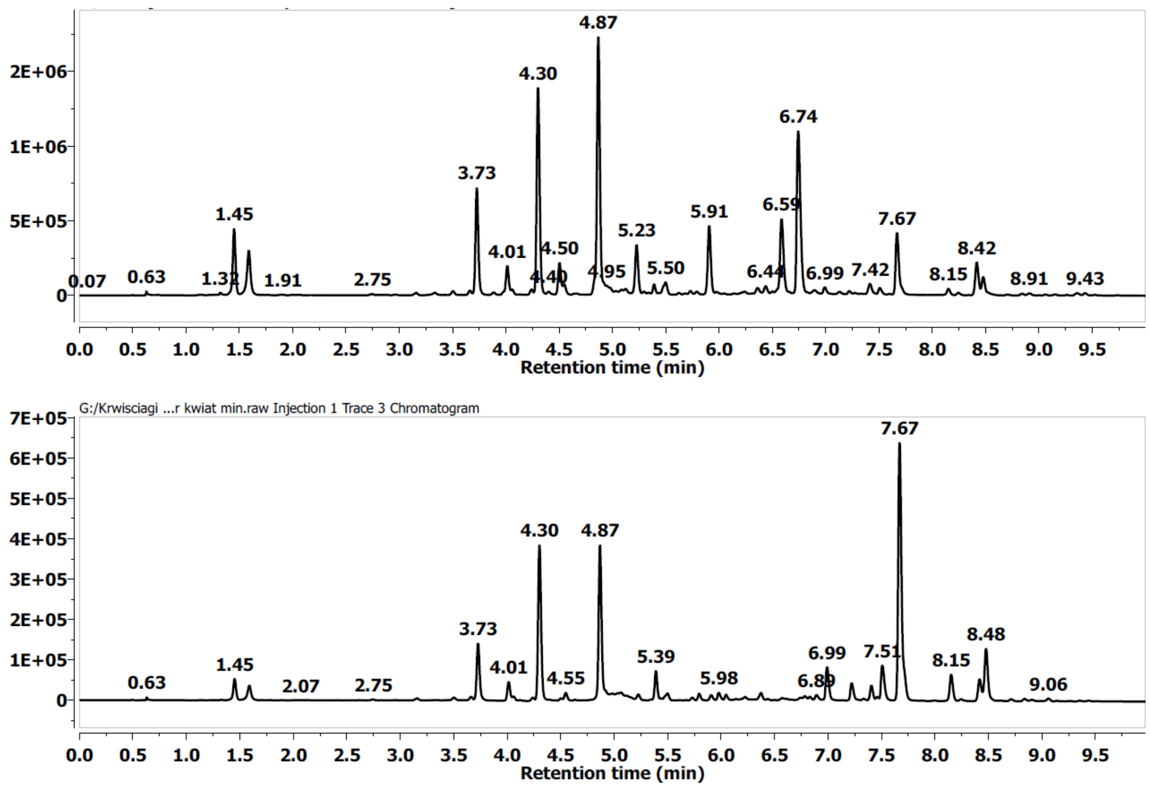

Figure S1. LC–DAD-ESI–QTOF–MS/MS chromatogram fragile of the *Sanguisorba officinalis* L. flowers extract at 320 and 360 nm.

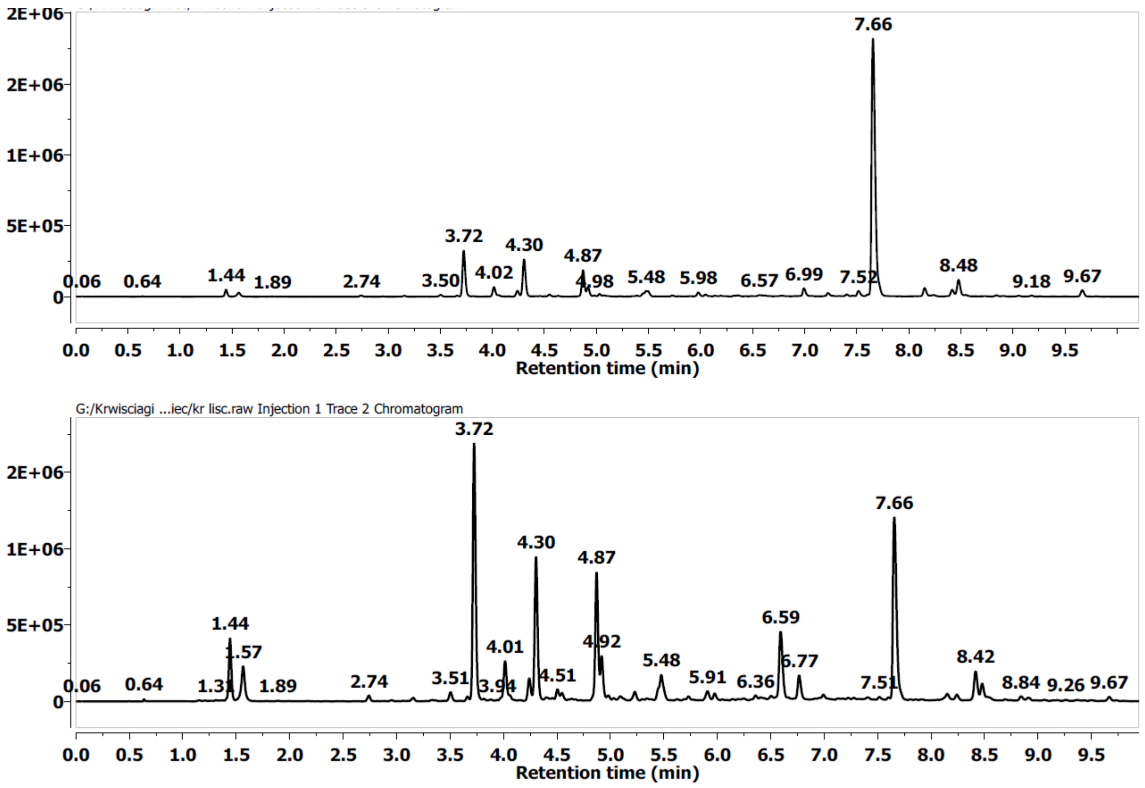

Figure S2. LC–DAD-ESI–QTOF–MS/MS chromatogram fragile of the *Sanguisorba officinalis* L. leaves extract at 320 and 360 nm.

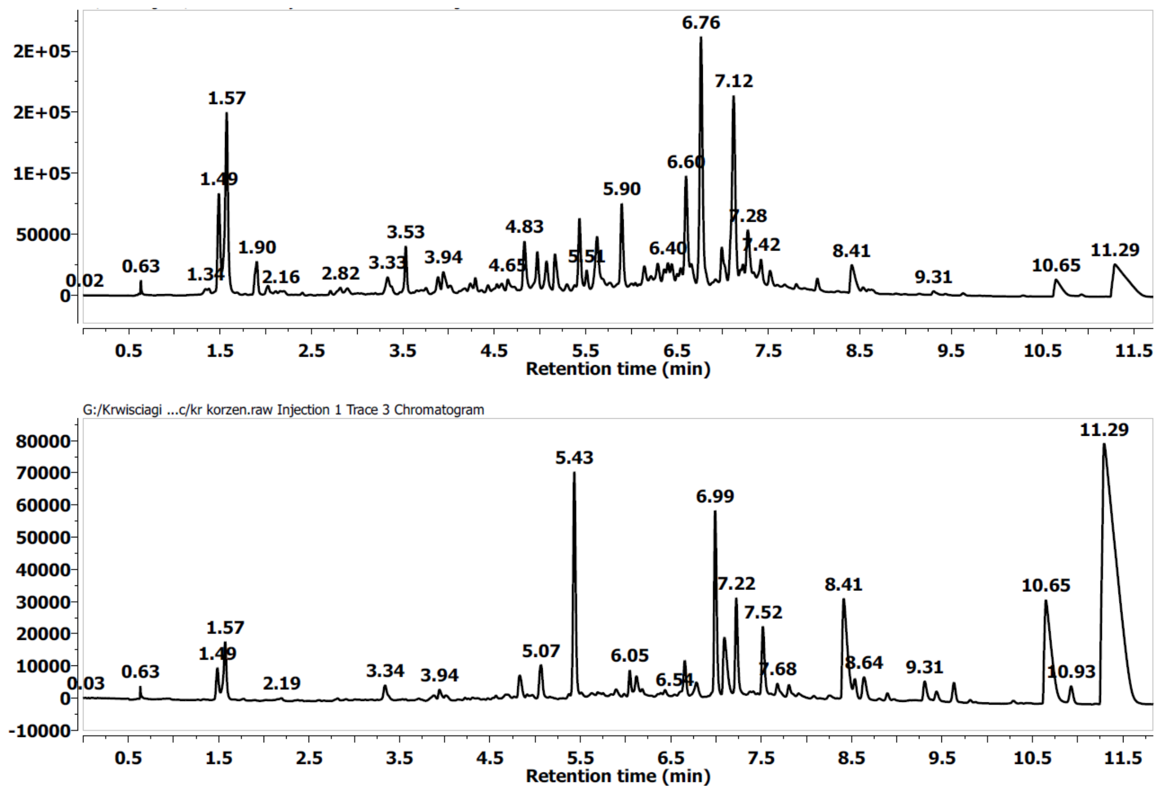

Figure S3. LC–DAD-ESI–QTOF–MS/MS chromatogram fragile of the *Sanguisorba officinalis* L. roots extract at 320 and 360 nm.

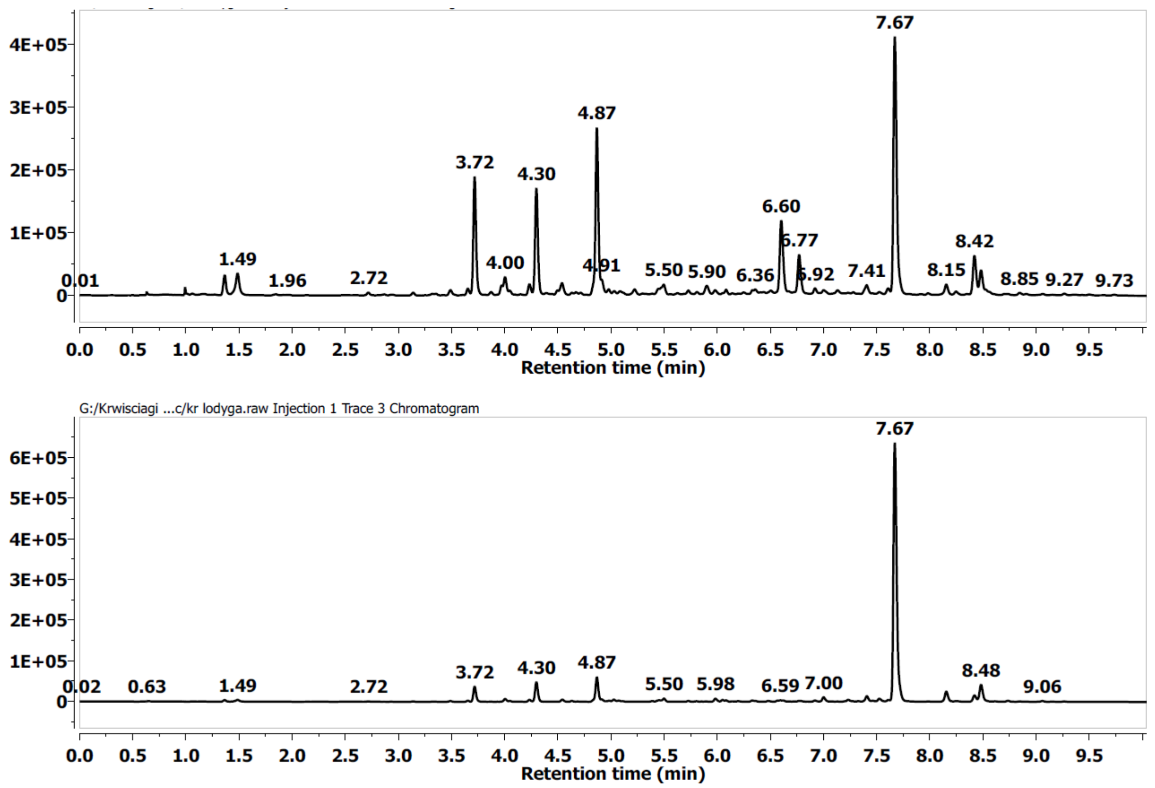

Figure S4. LC–DAD-ESI–QTOF–MS/MS chromatogram fragile of the *Sanguisorba officinalis* L. stalks extract at 320 and 360 nm.
